# Military Notes

**Published:** 1918-05

**Authors:** 


### Military Notes.

8% of the wounds of the Civil War involved the thorax, the mortality being 27.8%. In the Spanish-American War, the mortality of wounds in the thorax was about 9.5%; and slightly less in the Boer War. This was due not only to the introduction of principles of antisepsis but to the smaller calibre and higher velocity of bullets. In the present war, the mortality has risen to 14.5% partly because the fighting has been on highly polluted soil, partly because the stationary nature of the fighting has enabled many very serious cases to be included among the wounded treated which, previously, would have died before collection was possible. The mortality of chest wounds differs widely if we distinguish between penetrating and non-penetrating. Over 50% of the flesh wounds of the Civil War were located in the chest, and the mortality was only 1%, whereas the mortality of penetrating wounds of the chest was 62.5% and, in the Crimean War, it was 91.5%.

Many chest wounds recover under expectant treatment. In a case in which it seemed certain that the right auricle was pierced, the soldier was back on the firing line within a few weeks. Although a reasonable attempt is made to extract projectiles, this is not always possible, and a bullet in the parenchyma of the lung does not necessarily prevent healing and restoration of health.

13 cases of bullets lodged in the heart have been treated in French hospitals during the present war, with 10 recoveries. The vast majority of such wounds are, as heretofore, almost immediately fatal, but French surgeons feel that as surgery is pushed to the front, in accordance with the present policy, the results in cases that are not immediately fatal from massive haemhorrage, will be very good, not even of so high a mortality as penetrating abdominal wounds.

It will be remembered that when the modern, high velocity, small bullet was first introduced, it was prophecied that the majority of wounds would be uninfected, that the foreign body would escape by its own momentum, and that the lesion would be comparatively slight. Experience showed, however, that while this was measurably true for distances beyond the first thousand yards or thereabouts and up to the distance when diminishing velocity allowed a wabbling of the missile, the tremendous velocity of the bullet at short range produced an extensive laceration of tissues, which has been termed an explosive action. For example, a bullet at short range will make a small hole through an empty can, but will "explode" a can filled with water. An army surgeon however, has pointed out that the term "explosive" is not strictly correct. The enormous momentum of a bullet at short range, meeting even a slight obstruction, is inevitably dispersed laterally, and in fact in all directions, in accordance with well known physical principles.

The objection to using iodine before suppuration has occurred, is that it coagulates albumin and lowers the vitality of the wound. Turpentine has been suggested as a substitute but, on the whole, properly prepared Dakin's solution has given the best results.

Contrary to the regulations of the U. S. army, any French officer is authorized to buy any special article of food that his command requires. It should be borne in mind, however, that the customary demands of the French are lower than would be the case among American troops. Meat, for example, is ordinarily issued only twice a week. Wine is also habitually used and, as it is very cheap, it is estimated that 30 centimes buys as many calories in wine as 2 francs in meat.

Tin containers have been discarded from the first aid packets in France on account of the increased severity of a wound if the packet is struck. Waxed paper has been substituted.

Among the special malingering devices detected are: use of picric acid to simulate jaundice; smoking cigarettes impregnated with sodium chlorid to acquire pallor; rubbing with sand to produce dermatitis.

While the wound of entry is usually smaller than that of exit, exceptions occur so that this rule cannot be relied upon to detect self-mutilation.

Shrapnel bullets, having a comparatively small initial velocity, usually do not inflict much traumatic damage, but are dangerous mainly from introducing infected soil and clothing. They may flatten against a bone without fracturing it, and may be stopped by the skin opposite the point of entry.

Bayonet wounds are rarely encountered in military practice, being usually fatal before the wounded can be brought even to the first aid station. For this reason, the British and Canadian soldiers prefer to use the bayonet whenever practicable. It is claimed that one man killed 28 Germans in rapid succession, as they were driven out of a cave. The principle of bayonet practice is the same as boxing, both offensively and defensively. Soft parts are chosen to prevent sticking of the bayonet in the wound and one British soldier, expert with the bayonet, being virtually disarmed because his bayonet was firmly lodged between the ribs of his adversery, extricated it by discharging his gun.

Statistics of abdominal wounds are somewhat fallacious.

Some series seem to show that even cases subjected to prompt operation do not give better results than those in which expectant treatment is used. Other series contradict this impression and the general opinion is being developed that the treatment should, so far as possible, be discriminating. As abdominal wounds are especially liable to prevent locomotion most such cases are brought in at night, when wounded can be sought without almost inevitable death of stretcher parties. In such cases, operation is often impossible on account of cold alone. It is sometimes difficult to discriminate between wounds of the abdominal wall and those which penetrate, usually impossible to determine whether a penetrating wound has injured one or more viscera or has passed between them. Even without visceral lesion, a penetrating wound may cause extensive haemorrhage or septic peritonitis. Those whose stomachs and intestines are relatively empty give better results than those wounded after they have eaten heartily, for obvious reasons. The aggregate mortality of abdominal wounds is nearly 70%—40% shock, 20% peritonitis, 10% haemorrhage. 64% mortality is the best report thus far made of any considerable series.

The soil of Belgium is loaded with anaerobic bacteria, including the tetanus and gas bacillus, from centuries of intensive cultivation. A combined serum is used as a routine in all wounds. Owing to the economic value of manure and the custom of building dwellings, barns, stables and outhouses in a hollow square, practically all available buildings in the country are infested with flies and the water is highly contaminated, though not necessarily with bacteria of specific human diseases. (Note: The French word ferme, from which our word farm is derived, means the square inclosure of buildings, not the land, the word being ultimately the same as **firm,** in allusion to the security of the inclosure.)

Any fairly blunt projectile of high velocity, practically always carries into the wound, all the layers of clothing, as smoothly cut as if by a paper punch. The clothing is almost always infected from soil and perspiration.

Wounds of the hands and buttocks practically always show gas bacillus infection, those of the face rather infrequently. If gas gangrene does not develop within 24 hours, the case is considered fairly safe on this score.

A series of 2203 wounds treated at a hospital in Belgium April-October, 1915, were classified as follows: rifle bullets 497, shrapnel 94, shell fragments, etc., 1074, accidents not directly due to combat 203, fractures 179, other wounds 108, undetermined or unspecified from imperfections of records 48. Over 75% of this series were returned to the firing line.

